# Protein Adsorption on a Multimodal Cation Exchanger: Effect of pH, Salt Type and Concentration, and Elution Conditions

**DOI:** 10.3390/molecules30163389

**Published:** 2025-08-15

**Authors:** Jana Krázel Adamíková, Monika Antošová, Tomáš Kurák, Milan Polakovič

**Affiliations:** Department of Chemical and Biochemical Engineering, Institute of Chemical and Environmental Engineering, Faculty of Chemical and Food Technology, Slovak University of Technology, Radlinského 9, 812 37 Bratislava, Slovakia; adamikova.ja@gmail.com (J.K.A.); monika.antosova@stuba.sk (M.A.); tomas.kurak@stuba.sk (T.K.)

**Keywords:** multimodal chromatography, protein adsorption, cation exchange, hydrophobic interactions, Hofmeister series, elution conditions, bovine serum albumin, lysozyme, fetuin

## Abstract

This study investigates key factors affecting the adsorption behavior of proteins on the multimodal chromatographic adsorbent Capto MMC, aiming to enhance selective protein separation strategies. Batch equilibrium experiments were conducted using six model proteins to explore the combined effects of pH, ionic strength, and the nature of salts (kosmotropic and chaotropic) on protein–ligand interactions. Given that the Capto MMC ligand supports multiple interaction modes beyond cation exchange, particular focus was placed on acidic proteins (pI 4–5), which exhibited binding even at moderately elevated pH values—conditions ineffective for conventional cation exchangers. Hydrophobic interactions were identified as critical for stable binding of proteins like BSA and fetuin, while hydrophilic proteins such as ovalbumin showed minimal adsorption. Chromatographic column experiments were performed to evaluate elution performance under various buffer conditions, revealing that prolonged adsorption phases can reduce recovery yields for proteins with less stable tertiary structures. The findings highlight how salt type, pH, and protein hydrophobicity interplay to modulate multimodal binding mechanisms, providing practical insights for the design of tailored purification protocols.

## 1. Introduction

Therapeutic proteins are among the most valuable pharmaceutical products today. Since they cannot be synthesized through purely chemical means, their production relies on living organisms, typically genetically modified cells cultivated via recombinant DNA technology [1]. These cells are grown under controlled conditions in bioreactors. However, the co-production of undesired proteins and metabolites is unavoidable. Following the upstream process, the target protein is present in a highly dilute and relatively impure solution. In some cases, its concentration in the supernatant is as low as 0.1–1 g/L, with purity often not exceeding a few percent [2].

Chromatography plays a central role in the downstream purification of these proteins. Conventional chromatographic techniques include ion exchange and hydrophobic interaction chromatography, as well as more selective methods such as affinity or immunoaffinity chromatography. While traditional adsorbents are more cost-effective, they typically lack the selectivity provided by affinity-based materials. Consequently, there is an ongoing interest in developing new adsorbents with improved performance-to-cost ratios.

Among these, multimodal adsorbents stand out due to their ability to engage in multiple interaction mechanisms simultaneously, thanks to their specially designed ligands. A key advantage of these adsorbents is their enhanced tolerance to salt, which makes them especially suitable for capturing proteins directly from harvested cell culture supernatants [3]. Additionally, the combination of interaction modes may allow a single multimodal column to replace multiple conventional columns, or to enable unique selectivity not achievable by other methods. For example, Molnár et al. utilized the adsorption properties of Capto MMC for the purification of recombinant human erythropoietin [4].

On the downside, the stronger and more complex binding interactions of multimodal adsorbents make elution more challenging. All attractive interactions must be simultaneously weakened to achieve effective protein desorption. While increasing the salt concentration in the buffer is sometimes sufficient, such cases are rare [5]. More often, effective elution requires a combination of pH adjustment and increased ionic strength [6,7], the addition of strongly chaotropic salt [8] or the use of mobile-phase modifiers such as ethylene glycol or arginine [8,9,10].

By appropriately selecting the adsorption conditions, multimodal adsorbents can be effectively employed for the capture of monoclonal antibody (mAb) aggregates [11,12,13,14]. Rupčíková et al. provide a comprehensive review of the use of multimodal adsorbents for the removal of aggregates [15]. The broad range of conditions under which high selectivity can be achieved makes multimodal adsorbents particularly well-suited for flow-through purification modes, where impurities are selectively retained on the adsorbent [12,16,17].

Overall, the mechanisms governing protein binding to multimodal adsorbents under varying conditions remain difficult to predict and interpret. Most multimodal ligands combine hydrophobic and ion-exchange functionalities—interactions that are often antagonistic. Across a wide range of conditions, both interaction types may contribute to protein binding; however, increasing salt concentrations generally weaken electrostatic interactions while enhancing hydrophobic effects. Compounding this complexity, proteins are large, flexible molecules with multiple functional groups and conformations that can shift in response to process conditions [18]. Together, these factors create a highly variable system that cannot be easily described by simple models.

As a result, a growing body of literature is dedicated to exploring the interaction mechanisms between various proteins and multimodal ligands. For example, Nfor et al. applied thermodynamic modeling to determine adsorption isotherms for five different proteins on four multimodal resins [19]. Hou and Cramer examined the affinity and selectivity of a multimodal anion exchanger, Capto Adhere, using a broad range of proteins [20]. Their study included linear NaCl gradient experiments and assessed the effects of mobile phase modifiers such as ethylene glycol, urea, and arginine. Similarly, Wolfe et al. investigated the role of such modifiers in wash buffers to develop selective separation methods on Capto MMC [21]. Gudhka et al. investigated the influence of temperature and salt concentration on antibody surface interactions in multimodal cation exchange chromatography [22].

Woo et al. published two notable studies on how ligand structure influences protein binding to multimodal adsorbents. The first explored geometric constraints, charge density, ligand pKa, and the role of polar substituents near hydrophobic groups [23]. The second compared Capto MMC and Nuvia cPrime, focusing on differences in their aliphatic and aromatic content [24]. Zhu and Carta studied the effects of pH and NaCl concentration on both adsorption equilibria and kinetics of lysozyme on Capto MMC and Nuvia cPrime [25].

Despite these valuable insights, the fundamental understanding of protein–multimodal ligand interactions remains incomplete. While it is generally accepted that salt type significantly influences hydrophobic interactions, systematic studies involving salts other than NaCl are still limited. For instance, Yang et al. examined the effect of MgC_2_ in lectin separation [26], while other studies explored the influence of (NH_4_)_2_SO_4_ on protein elution [21,27]. Kreusser at all. studied the adsorption of lysozyme on Toyopearl MX-Trp-650M using four salts: sodium chloride, sodium sulfate, ammonium chloride, and ammonium sulfate. [28]. More recently, Altern et al. employed mechanistic models based on high-throughput batch isotherm data to discriminate between isotherm models for monoclonal antibody adsorption on Capto MMC and Capto Adhere, considering the effects of pH and salt type [29].

This study bridges the fundamental insights into protein–ligand interactions with the development of a practical workflows for preparative protein separation using a multimodal chromatographic adsorbent. Six model proteins with diverse physicochemical properties were selected to investigate how pH, ionic strength, and salt type (kosmotropic vs. chaotropic) influence binding mechanisms. Capto MMC, previously identified as the most effective capture-step adsorbent for recombinant human erythropoietin [4], was employed as the model chromatographic adsorbent in this study.

Particular focus was placed on the adsorption behavior of proteins with isoelectric points between pH 4 and 5 at elevated pH levels—conditions under which conventional cation exchangers are typically ineffective. Recognizing the unique multifunctionality of the Capto MMC ligand, we explored the contribution of hydrophobicity to the binding of acidic proteins, aiming to delineate interaction patterns beyond simple ion exchange. The experimental approach combined batch adsorption studies for equilibrium characterization with chromatographic column experiments designed to assess elution strategies, adsorption kinetics, and gradient effects on protein recovery, providing a comprehensive framework for tailoring purification protocols.

## 2. Results and Discussion

The adsorption behavior of proteins with varying properties on the multimodal adsorbent Capto MMC was first investigated through a series of equilibrium batch adsorption experiments. Compared to column chromatography, batch adsorption is significantly less material- and time-intensive and was therefore selected as the initial method in this study.

### 2.1. Batch Equilibrium Experiments

#### 2.1.1. Effect of pH and NaCl Concentration

Capto MMC is fully protonated at pH 3, making electrostatic interactions between proteins and its ligand negligible, while hydrophobic interactions dominate [25]. Zhu and Carta demonstrated that both lysozyme and a monoclonal antibody (mAb) with a pI of 8 could bind with high capacity under these conditions, and that binding capacity slightly increased with NaCl concentration [25]. The same authors found that at pH 7, Capto MMC is almost completely deprotonated, and its adsorption behavior resembles that of a conventional cation exchanger. At this pH, the mAb bound significantly only in the absence of NaCl, with binding capacity decreasing sharply as NaCl concentration increased. In contrast, lysozyme binding at pH 7 was partially driven by hydrophobic interactions, and its binding capacity decreased less dramatically with increasing NaCl concentration.

**Figure 1:**
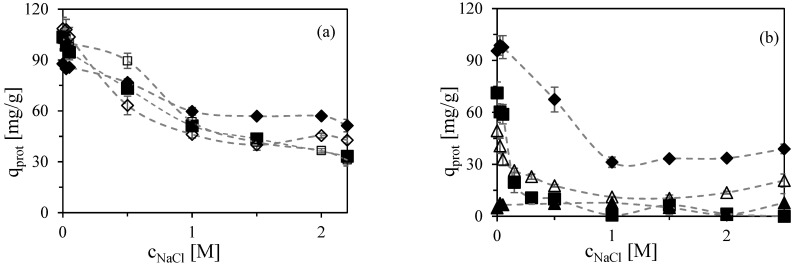
Influence of NaCl concentration on specific equilibrium of adsorbed amount of protein on multimodal adsorbent Capto MMC at (**a**) pH 5 and (**b**) pH 6. Symbols correspond to individual proteins: ■ BSA, ☐ HSA, ◆ LYZ, ◇ CYGB Ab, ▲ OVA, △ FET. Error bars represent standard deviations.

Based on this knowledge, we have focused on pH 5 and 6 in this study. The specific adsorbed amounts of proteins obtained under equilibrium conditions at these two pH values are presented in Figure 1. The uncertainty of the values, as determined from duplicate experiments, was less than 10%. At pH 5 and in the absence of added NaCl, the adsorbed amounts for all tested proteins ranged from 90 to 110 mg/g, aligning well with previously published values [25,30]. These similar adsorption capacities were observed despite differences in protein properties, such as net charge. For instance, at pH 5, bovine serum albumin (BSA) and human serum albumin (HSA) are near their isoelectric points and thus expected to exhibit minimal net surface charge, whereas lysozyme and cytoglobin antibody—with higher isoelectric points—carry a net positive charge.

The adsorption of BSA and HSA at pH 5 is common on conventional cation exchangers [31,32]. Adsorption of proteins with near-zero net charge is here typically attributed to the heterogeneous distribution of charged amino acid residues on the protein surface or to the so-called Donnan effect. This phenomenon assumes the formation of an electrical double layer at the positively charged adsorbent–liquid interface, which attracts protons while excluding negatively charged ions [19]. Consequently, the local pH near the adsorbent surface can be up to one unit lower than the bulk solution pH, facilitating protein binding even under seemingly neutral conditions.

The data presented in Figure 1 confirm that at pH 5 and 6, hydrophobic and electrostatic interactions act cooperatively, resulting in relatively high protein-binding capacities and increased salt tolerance [25]. Although the amount of adsorbed proteins decreased with increasing NaCl concentration due to the impact of electrostatic interactions, the persistence of significant protein adsorption even at high ionic strength (Figure 1a) clearly indicates the contribution of hydrophobic interactions.

At a NaCl concentration of 2.2 M and pH 5, the adsorbed amounts of BSA and HSA remained at approximately 30% of their initial levels (in salt-free solution), cytoglobin antibody at 40%, and lysozyme at 60%. These findings are consistent with the results reported by Woo et al., where both BSA and lysozyme were retained on Capto MMC across the entire NaCl concentration range of 0–1.5 M [24]. This behavior contrasts sharply with conventional ion exchangers, which typically lose their protein-binding capability at NaCl concentrations as low as 200–300 mM [33].

At a higher pH of 6, BSA becomes negatively charged and, therefore, should not bind to a cation exchange adsorbent due to electrostatic repulsion. Indeed, several studies report that BSA fails to bind to conventional cation exchange resins at pH 6 or higher [31,34]. However, BSA is known to bind to multimodal cation exchangers, such as Capto MMC, under these conditions [19,35]. As shown in Figure 1b, the adsorbed amount of BSA at pH 6 in the absence of NaCl was around 70 mg/g. A similar BSA binding profile has been observed even at pH 7 [36].

This binding behavior may be attributed to hydrophobic interactions, which facilitate protein orientation and approach to the adsorbent pore surface. Although these interactions alone may not drive binding at low salt concentrations, they allow BSA—containing positively charged residues in proximity to hydrophobic regions—to interact effectively with negatively charged ligands. It has been suggested that the N-terminal region of BSA, rich in basic and hydrophobic amino acids (particularly arginine), plays a key role in this process [37].

In the case of lysozyme, its adsorption onto Capto MMC at pH 6 remained similar to that at pH 5 (Figure 1b), which was expected given its high isoelectric point. The lysozyme titration curve indicates only minor changes in net charge across this pH range, enabling consistent binding to cation exchange ligands [19]. Furthermore, lysozyme adsorption on hydrophobic adsorbents has also been shown to be pH independent [38].

A comparison of two glycosylated proteins—ovalbumin and fetuin—revealed distinct adsorption behaviors (Figure 1b). Although both proteins have low isoelectric points and are negatively charged at pH 6, their different glycosylation patterns significantly affect their hydrophobicity. Fetuin, with a high content of sialic acid residues, exhibits increased hydrophobic character, while glycopeptides rich in mannose residues, such as ovalbumin, are more hydrophilic [39]. As a result, ovalbumin was not retained by Capto MMC at pH 6, which aligns with the literature findings [24]. In contrast, fetuin was adsorbed under low-salt conditions. Similarly to BSA, this behavior can be attributed to hydrophobic interactions that orient the protein in a way that allows positively charged patches to interact with negatively charged adsorbent groups, even at pH values above the protein’s pI.

Another key observation at pH 6 was a reduction in the salt tolerance of Capto MMC. At NaCl concentrations above 0.3 M, BSA was not adsorbed, and lysozyme adsorption was also reduced. Similar results were reported by Woo et al. [24], who noted that at pH 5, neither BSA nor lysozyme could be eluted with high salt concentrations. However, at pH 6, BSA was eluted at 0.3 M NaCl and lysozyme at 1.2 M, suggesting that increasing pH weakens the strength of hydrophobic and electrostatic interactions, thereby lowering overall salt tolerance.

#### 2.1.2. Effect of Salt Type and Concentration

To further investigate the role of hydrophobic interactions in multimodal chromatography, the effect of different salt types was examined. It is well established that salts exert a significant influence on hydrophobic interaction chromatography (HIC), and their application in multimodal systems has been previously reported for protein separation [8,40]. However, to the best of our knowledge, no systematic study has been published on the effect of salt type in multimodal chromatography using model proteins. Such data could contribute to a more generalized understanding of the underlying binding mechanisms and interaction phenomena.

**Figure 2:**
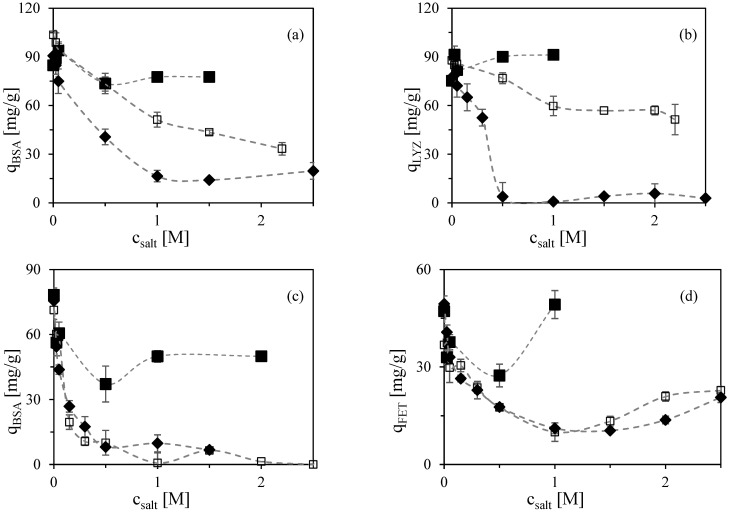
Effect of salt concentration on the specific equilibrium of adsorbed amount of protein on the multimodal adsorbent Capto MMC: (**a**) BSA at pH 5, (**b**) LYZ at pH 5, (**c**) BSA at pH 6, and (**d**) FET at pH 6. Salt types are distinguished by symbols: ■ (NH_4_)_2_SO_4_, ☐ NaCl and ◆ MgCl_2_. Error bars represent standard deviations.

In this study, three salts representing different positions in the Hofmeister series were selected: a strong kosmotropic salt, ammonium sulfate ((NH_4_)_2_SO_4_), which promotes hydrophobic interactions; a mildly kosmotropic salt, sodium chloride (NaCl); and magnesium chloride (MgCl_2_), which exhibits chaotropic behavior mainly at low concentrations [41]. However, MgCl_2_ decreases protein solubility at pH 4.5–5 and above, so a transition from salting-in to salting-out occurs with increasing concentration of this salt [41]. The influence of these three salts on the adsorption of BSA, lysozyme, and fetuin at pH 5 and pH 6 is shown in Figure 2.

At pH 5 (Figure 2a,b), the observed effects align well with the Hofmeister series. The presence of (NH_4_)_2_SO_4_ induced strong hydrophobic binding that effectively compensated for the loss of electrostatic interactions at higher salt concentrations. As a result, the adsorbed amounts of both BSA and lysozyme remained relatively constant across the full range of ammonium sulfate concentrations tested. This finding suggests that a high concentration of (NH_4_)_2_SO_4_ in the loading buffer does not inhibit protein binding to the Capto MMC ligand. However, it also implies that such a buffer is not suitable for protein elution, as the hydrophobic interactions are too strong to be disrupted under these conditions [21]. Conversely, MgCl_2_, as a chaotropic salt, interfered with hydrophobic interactions and caused a more pronounced decrease in protein adsorption. In particular, lysozyme failed to bind at MgCl_2_ concentrations above 0.5 M—an effect that was not observed with NaCl, even at comparable or higher concentrations. This confirms the destabilizing effect of chaotropic agents on hydrophobic interactions within the multimodal binding mechanism.

At pH 6 (Figure 2c,d), the effect of salt type exhibited a somewhat different pattern compared to pH 5. Specifically, in the case of (NH_4_)_2_SO_4_, the transition from electrostatic to hydrophobic binding mechanisms was marked by a distinct minimum in the adsorbed protein amount. This characteristic U-shaped adsorption profile is commonly observed when the dominant binding mechanism in multimodal adsorbents shifts with changing ionic strength [19]. Initially, as the concentration of (NH_4_)_2_SO_4_ increases, ionic interactions are suppressed, leading to a decline in protein adsorption. However, at higher salt concentrations, hydrophobic interactions become dominant, resulting in a recovery and even increase in the adsorbed protein amount. This behavior reflects the dual binding nature of multimodal ligands and the competitive influence of salt on different interaction forces.

A comparable trend was reported by Creasy et al., who observed a U-shaped dependence of lysozyme binding affinity on hydrophobic interaction chromatography resins in the presence of ammonium sulfate [42,43]. Their findings support the hypothesis that salt-induced modulation of binding modes is a general phenomenon, applicable across different types of chromatographic media with multimodal characteristics.

A different behavior was observed in the presence of NaCl and MgCl_2_. Both salts exhibited a similar effect on protein adsorption, significantly reducing the adsorbed amounts of BSA and fetuin. However, the adsorbed amount of fetuin remained above 10 mg/g across all tested conditions. This residual binding is likely attributable to the strong hydrophobic character of the fetuin molecule. This suggests that for highly hydrophobic proteins, hydrophobic interactions can maintain binding affinity despite the disruption of electrostatic interactions. In case of MgCl_2_, the enhanced fetuin adsorption at the concentrations above 1 M can be explained by the abovementioned transition from salting-in to salting-out conditions [41].

### 2.2. Chromatographic Column Experiments

#### 2.2.1. Frontal Experiments

**Figure 3:**
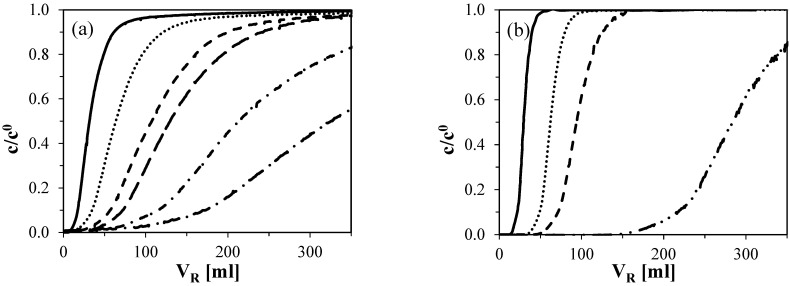
Influence of (**a**) BSA and (**b**) LYZ concentration on breakthrough curve shapes on Capto MMC in 50mM acetate buffer at pH 5. The slope of the curves decreases with decreasing initial protein concentration. The initial concentrations are distinguished by line styles: 1 g/L—solid line (—), 0.5 g/L—dotted (...), 0.3 g/L—short dashed (- - -), 0.25 g/L—long dashed (– – –), 0.15 g/L—dash-dotted (- . -), and 0.1 g/L—dash-double dotted (– . . –).

In subsequent experiments, protein interactions with the multimodal Capto MMC adsorbent were further investigated using column chromatography. Breakthrough curves for BSA and lysozyme at various initial protein concentrations are presented in Figure 3. The adsorption buffer was selected based on the outcomes of the batch equilibrium experiments, with the aim of maximizing protein binding. Accordingly, a 50 mM acetate buffer at pH 5 was employed, as it yielded the highest adsorbed protein amounts under batch conditions.

As shown in Figure 3a, the BSA breakthrough curves exhibit pronounced tailing, meaning that it takes a prolonged period for the BSA concentration at the column outlet to reach the inlet concentration. This behavior has previously been reported by Hunter and Carta in the context of conventional anion-exchange chromatography [44]. They attributed the tailing effect to the presence of BSA dimers or higher-order oligomers commonly found in standard BSA preparations, which possess a higher affinity for the adsorbent compared to monomeric BSA [45]. Notably, even when BSA was adsorbed at a higher NaCl concentration of 0.5 M (data not shown), the tailing behavior persisted, suggesting that this effect is independent of ionic strength within the tested range. In contrast, no such tailing was observed for lysozyme, as illustrated in (Figure 3b), indicating a more uniform interaction with the Capto MMC ligand.

**Figure 4:**
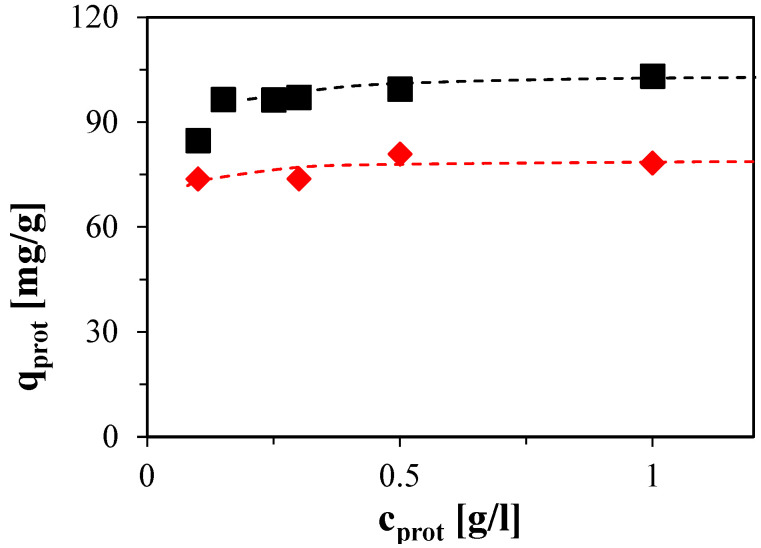
Adsorption isotherms for BSA (■) and lysozyme (◆) on Capto MMC in 50 mM acetate buffer at pH 5. Dashed lines represent maximum binding capacity.

The adsorption isotherm points, shown in Figure 4, were determined by integrating the outlet protein concentration from the breakthrough curves. Under conditions favorable for protein adsorption, the resulting isotherms exhibit a nearly rectangular shape. This suggests strong binding to the adsorbent. Notably, even at an equilibrium protein concentration as low as 0.1 g/L, the amount of adsorbed protein approached the maximum binding capacity of the respective protein, indicating high affinity and efficient utilization of the binding sites.

#### 2.2.2. Elution Experiments

In the next series of experiments, the focus was placed on the effect of elution conditions on desorption efficiency. To conserve materials, a column load of 10% was employed. BSA and fetuin were selected as model proteins to explore methods for separating acidic proteins with differing hydrophobic characteristics. The adsorption buffer composition was chosen based on batch equilibrium adsorption results to maximize protein binding. Specifically, BSA was adsorbed using 50 mM acetate buffer at pH 5, while fetuin was adsorbed at pH 6 without additional salt. Although pH 5 might be more convenient for fetuin as well, this pH is close to its isoelectric point, causing low protein solubility.

**Figure 5:**
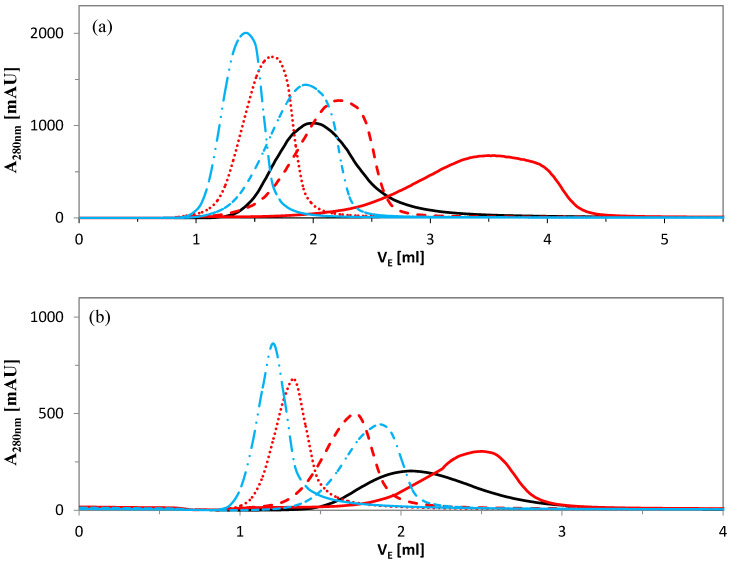
Influence of eluent on elution of (**a**) BSA and (**b**) fetuin. Buffer types are distinguished by color and pH by a line type. Acetate pH 6—solid line (—), Bis–Tris (red): pH 6—solid line (—), pH 6.5—short dashed (- - -), pH 7—dotted (····), Tris–HCl (blue): pH 8—dash-dotted (- · -), pH 9—dash-double dotted (— · · —).

Various elution conditions were tested. Elution curves for both proteins are shown in Figure 5 and the corresponding yields of BSA and fetuin are summarized in Table 1. Elution of BSA using only increasing NaCl concentration without pH adjustment (Eluent No. 1), proved largely ineffective (data not shown in Figure 5). Even after applying 16 BV of the buffer, the BSA elution curve did not approach the baseline and the BSA yield was only about 15% (Table 1). This suggests that disrupting electrostatic interactions alone was insufficient for complete BSA desorption, as hydrophobic interactions remained prominent. These findings are consistent with the static binding capacity data shown in Figure 1a.

To achieve efficient desorption, the pH was varied from 6 to 9 (Table 1), using different buffer types to ensure adequate buffering capacity across this range. Increasing the pH was expected to deprotonate the ligand’s ionic groups, resulting in both the ligand and the proteins carrying negative charges, which would induce electrostatic repulsion. Additionally, the increased negative charge on the proteins reduces their hydrophobicity, thereby weakening hydrophobic interactions with the ligand.

Figure 5 shows that the elution volume varies depending on the elution buffer used. Surprisingly, elution volume does not decrease simply with increasing pH; rather, it depends on the specific combination of buffer type, pH, and protein. The elution volume of BSA increases in the following order: Tris-HCl, pH 9 < Bis-Tris, pH 7 < Tris-HCl, pH 8 ≈ acetate, pH 6 < Bis-Tris, pH 6.5 < Bis-Tris, pH 6. For fetuin, the elution strength ranking that leads to the shortest elution volume differs slightly: Tris-HCl, pH 9 < Bis-Tris, pH 7 < Bis-Tris, pH 6.5 < Tris-HCl, pH 8 < acetate, pH 6 < Bis-Tris, pH 6.

At pH 6, the acetate buffer acts as a stronger eluent than Bis-Tris buffer for both proteins. Likewise, the Bis-Tris buffer with pH 7 is a stronger eluent than the Tris-HCl with pH 8. Evidently, the less polar and more hydrophobic Bis-Tris molecule, being two hydroxyethyl chains larger than the Tris molecule, eliminates hydrophobic interactions between the ligand and proteins more effectively. This effect outweighs the effect of enhanced electrostatic repulsion.

Additionally, the buffer type strongly influences the shape of the elution peak. Bis-Tris at pH 6 produced fronting, asymmetric peaks characteristic of anti-Langmuirian behavior (red lines in Figure 5a,b), while acetate buffer at pH 6 resulted in earlier elution with more symmetrical peaks (black lines in Figure 5a,b). Bis-Tris is the largest cation among the buffers tested and likely participates in nonspecific interactions with either the adsorbent or the proteins. BSA eluted significantly later than fetuin in the presence of Bis-Tris at pH 6. This may indicate stronger nonspecific interactions or, alternatively, conformational changes in desorbed BSA in the presence of this eluent.

Although the elution volume varies significantly, overall protein yield is similar across the experiments. Table 1 shows that combining an increase in both pH and NaCl concentration during elution significantly improved the recovery of BSA. When the pH was raised to 6 using acetate buffer, the BSA yield increased to approximately 90%, with complete protein desorption achieved at this higher pH. These findings align well with equilibrium adsorption results, where BSA remained strongly bound at pH 5 even at high NaCl concentrations, but binding was abolished at pH 6 when the NaCl concentration exceeded 0.5 M.

In contrast fetuin—containing a high amount of sialic acids—exhibited notably different elution behavior. Fetuin yields under the tested elution conditions ranging from 75% to 90%, with only a slight increase in yield observed as pH increased. The relatively lower yield of fetuin during chromatographic experiments is consistent with batch adsorption results, where a portion of fetuin remained bound to the adsorbent under all tested conditions.

To further investigate the impact of adsorption time, an additional experiment was conducted. After protein adsorption and column washing, the process was paused, and elution was initiated the following day—after a 16 h delay. The elution buffer used was 50 mM acetate at pH 6 with 2 M NaCl. Under these conditions, the BSA yield dropped significantly to 46%. This decrease suggests that adsorption is likely a multi-step process; some phases develop over time, resulting in stronger and potentially irreversible binding of BSA.

This phenomenon is likely related to hydrophobic interactions characteristic of multimodal adsorbents, as irreversible two-step adsorption is well-documented in hydrophobic chromatography [38,46,47,48,49]. In the first rapid step, proteins reversibly bind to the hydrophobic surface, while the second step likely involves protein unfolding and the formation of multiple strong binding interactions, which may not be reversible [46,48].

The reduced yield observed during delayed desorption (incubation) may hypothetically be caused by conformational changes in the bound proteins and their aggregation on the adsorbent surface. The affinity of the aggregates is higher due to hydrophobic interactions, which results in a lower yield. Such behavior was observed by Roberts and Carta during the adsorption of BSA on a multimodal anion exchanger [50,51].

Similar results were observed when modifying the transition from adsorption to elution buffer. In earlier experiments an abrupt mobile phase change was employed. However, when concentration of salt in the elution buffer was increased gradually by 10% every five bed volumes, the BSA yield remained low (approximately 47%.) This may be attributed to conformational changes in BSA during the slow buffer transition.

In contrast, fetuin did not exhibit these effects. Its yield remained consistent regardless of the extended adsorption phase or the gradual elution buffer change. Minor differences observed are likely due to experimental variability. Fetuin is not a standard model protein, and data on its behavior during hydrophobic adsorption or structural changes are limited. It is plausible that fetuin’s glycan moieties provide steric shielding, reducing its interaction with hydrophobic surfaces. Additionally, fetuin may possess a stable tertiary structure that resists significant conformational changes during adsorption and desorption processes. The observed consistent elution behavior likely results from a combination of these factors.

## 3. Materials and Methods

### 3.1. Materials

**Figure 6:**
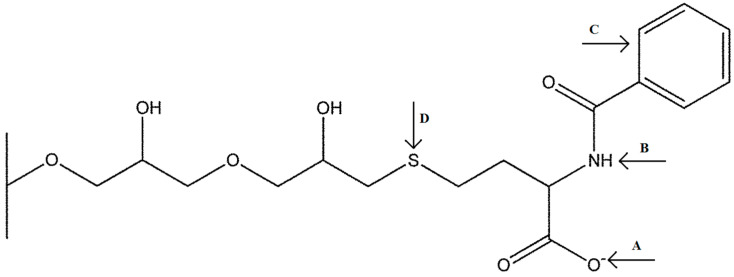
Ligand of multimodal adsorbent Capto MMC: N-benzoyl-homocysteine. The ligand can bind protein by ionic interactions (A), hydrogen bonds (B), hydrophobic interactions (C), and tiophilic bridges (D).

The multimodal adsorbent Capto MMC was obtained from Cytiva (Marlborough, MA, USA). Chemical structure of its ligand is shown in Figure 6. According to the manufacturer, the static binding capacity of the adsorbent is 45 mg/mL for bovine serum albumin (BSA).

The following proteins were purchased from Sigma-Aldrich (St. Louis, MO, USA): bovine serum albumin (BSA), human serum albumin (HSA), lysozyme (LYZ), cytoglobin antibody (CYGB Ab), ovalbumin (OVA), and fetuin (FET). The physicochemical characteristics of these proteins are summarized in Table 2. All other chemicals used were of analytical grade.

### 3.2. Static Batch Equilibrium Experiments

A 50 mM acetate buffer at pH 5 or 6, supplemented with varying concentrations of salts, was used as the adsorption buffer. The concentrations of NaCl, MgCl_2_, and (NH_4_)_2_SO_4_ were adjusted from 0 to 2.5 M. The initial protein concentration in the solution was 2 g/L. Pre-equilibrated Capto MMC adsorbent was added to the protein solution at a ratio of 33 mg/mL and mixed for 6 h to reach adsorption equilibrium. After equilibration, the protein concentration in the supernatant was measured spectrophotometrically at 280 nm. The specific amount of adsorbed protein $q_{prot}$, was calculated using a mass balance approach. Experiments were conducted in duplicate. For each adsorption experiment, a parallel control experiment without adsorbent was performed to account for protein loss due to factors other than adsorption.

### 3.3. Dynamic Column Chromatographic Experiments

Chromatographic experiments were conducted using an ÄKTA FPLC system (GE Healthcare, Uppsala, Sweden) operated with Unicorn 5.31 software (GE Healthcare, Uppsala, Sweden). A Tricorn 5/50 column (inner diameter 5 mm) packed with Capto MMC was used. The packed bed volume (BV) was 0.51 mL. The mobile phase volumetric flow rate was set to achieve a superficial velocity of 2.55 cm/min. Breakthrough curves were obtained for BSA and lysozyme using feed solutions at protein concentrations ranging from 0.1 to 1.0 g/L in 50 mM acetate buffer at pH 5. The specific adsorbed amount, $q_{prot}$, was determined from the mass balance of the breakthrough curve.

Elution conditions for BSA and fetuin were also investigated. BSA was adsorbed using 50 mM acetate buffer at pH 5, while fetuin was adsorbed at pH 6. The column was loaded with a protein amount corresponding to 10% of its equilibrium binding capacity at the feed concentration of 2 g/L. After adsorption, the column was washed with 3 BV of adsorption buffer, eluted with 16 BV of elution buffer and regenerated with 8 BV of 1M NaOH. Detailed elution protocols are given in the Results and Discussion section.

## 4. Conclusions

This study characterized the adsorption behavior of the multimodal adsorbent Capto MMC, which features a cation-exchange ligand. Both batch equilibrium and chromatographic column experiments were conducted, focusing on the effects of pH and salt concentration, including salts with differing kosmotropic and chaotropic properties. The results demonstrate that protein–ligand interactions are significantly influenced by salt type and pH highlighting the potential to achieve selectivity in chromatographic purification.

It was shown that certain acidic proteins, such as BSA and fetuin, can bind to Capto MMC at low ionic strength even at pH 6—a condition where conventional cation exchangers fail. Hydrophobic interactions are likely essential for orientating proteins correctly on the adsorbent surface to form stable binding. In contrast, ovalbumin, despite having a similar isoelectric point but greater hydrophilicity, did not bind to Capto MMC at pH 6.

Elution conditions were established, yielding high recoveries of BSA and fetuin. However, a gradual shift in mobile phase properties or prolonged adsorption times led to a marked decrease in BSA recovery, suggesting that proteins with less stable tertiary structures are more prone to irreversible binding under such conditions.

These findings are specific to the selected set of model proteins and to the Capto MMC adsorbent. Nevertheless, the study provides valuable insights into the interplay of electrostatic and hydrophobic interactions in multimodal adsorption systems.

## Figures and Tables

**Table 1:** Results of elution experiments with model proteins BSA and fetuin.

Eluent	Elution Conditions			Yield * [%]	
No.	Buffer Type	pH	c_NaCl_ [M]	BSA	Fetuin
1	Acetate	5	2	15	
2	Acetate	6	2	90	80
3	Bis–Tris	6	2	98	78
4	Bis–Tris	6.5	2	99	82
5	Bis–Tris	7	2	96	82
6	Tris–HCl	8	2	100	85
7	Tris–HCl	9	2	96	88

* The yield is related to the adsorbed protein amount.

**Table 2:** Characteristics of used model proteins.

Protein	Size [kDa]	pI Value	Glycosylation
BSA	66.4	4.7–5.3	no
HSA	66.4	4.7	no
LYZ	14.3	11.4	no
CYGB Ab	20.9	6	no
OVA	42.7	4.5	54% (*w*/*w*), mainly mannoses [52]
FET	48.4	3.5–4.2	26% (*w*/*w*), mainly sialic acids [53]

## Data Availability

Data will be made available on request from the corresponding author.
